# Supplementary material for: Development and Upscaling of SiO2@TiO2 Core-Shell Nanoparticles for Methylene Blue Removal
Source: Nanomaterials (Basel). 2023 Aug 8;13(16):2276. doi: 10.3390/nano13162276 (PMC10458987; doi:10.3390/nano13162276)
Supplement: Supplementary file 1 [file nanomaterials-13-02276-s001.zip › nanomaterials-2509642-supplementary.pdf]

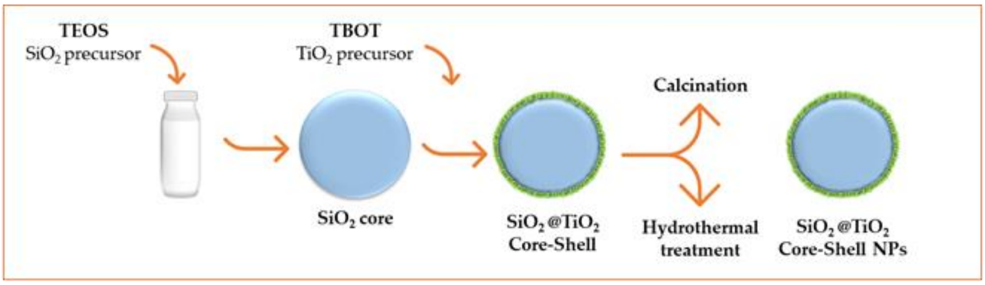

Figure S1. Main steps involved in the synthesis of the SiO2@TiO2 core-shell NPs.

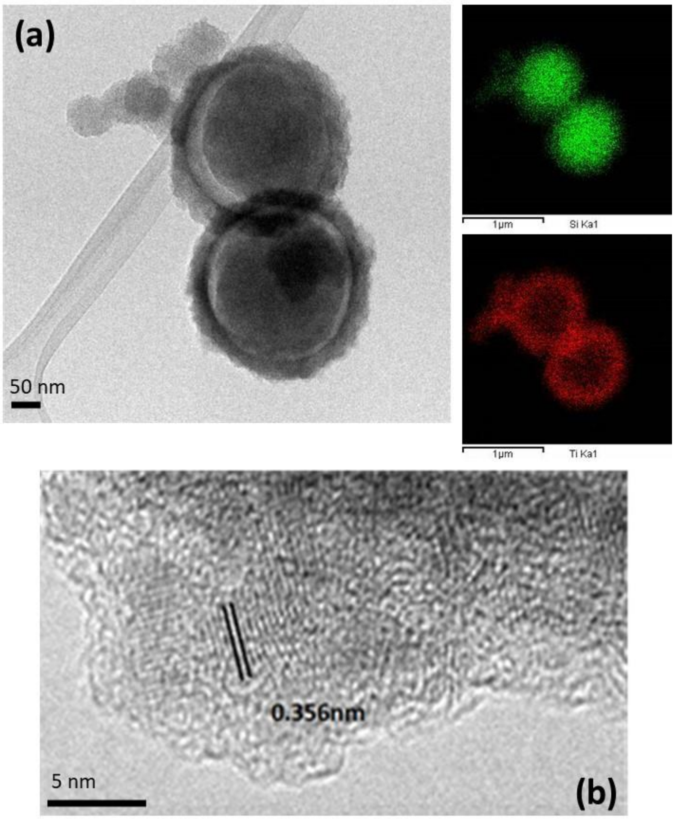

*Figure S2. HR-TEM images of (**a**) SiO2@TiO2 core-shell NPs HT1 and EDS Si (green) and Ti (red) element map of sample HT1. The detail in section (**b**) is the high-resolution image of the HT1 shell with the evidence of the 0.356 nm fringe pattern.*

Table S1. Images of the vials containing the powdered sample and the MB solution with different times of exposure to UV light and after filtration

<table>
<tr><th></th><th></th><th></th><th colspan="4">SiO_2@TiO_2</th><th></th></tr>
<tr><th></th><th>SiO_2</th><th>TiO_2</th><th>Calcined</th><th>HT1</th><th>HT2</th><th>HT1 US[1]</th><th>MB</th></tr>
<tr><td>0 min</td><td colspan="7">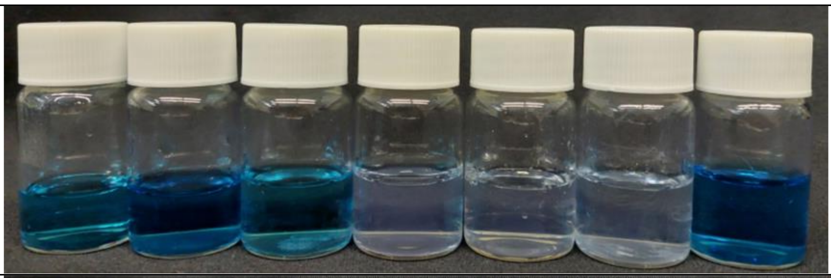</td></tr>
<tr><td>15 min</td><td colspan="7">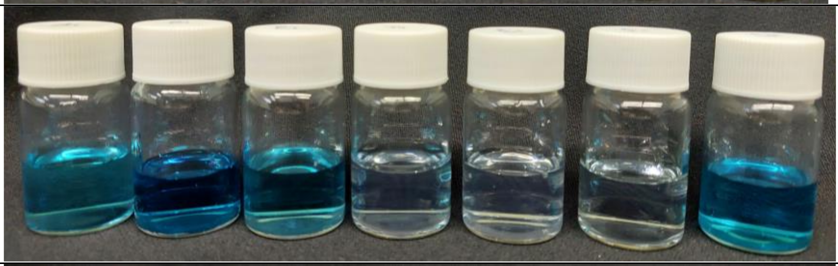</td></tr>
<tr><td>60 min</td><td colspan="7">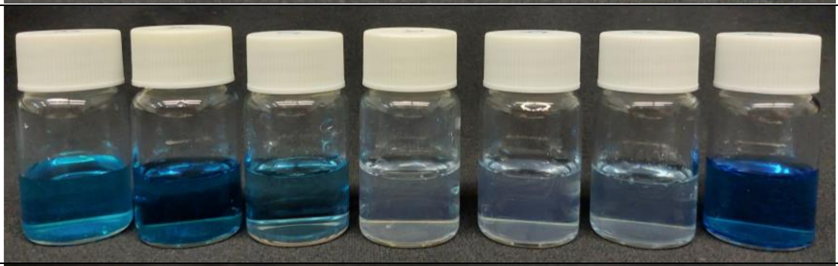</td></tr>
<tr><td>300 min</td><td colspan="7">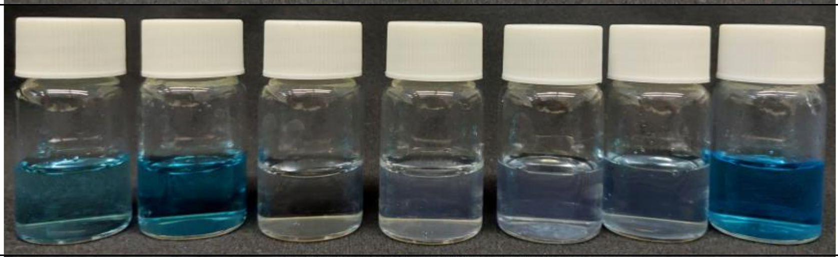</td></tr>
<tr><td>480 min</td><td colspan="7">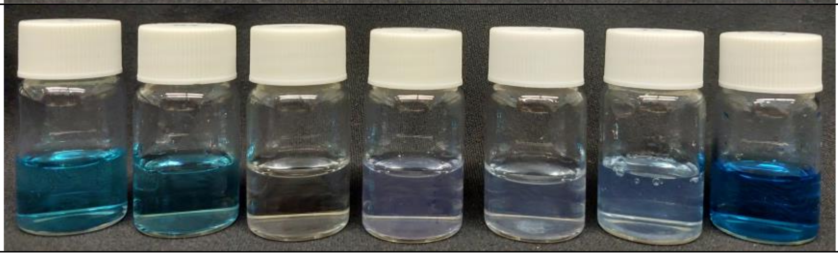</td></tr>
</table>

[1] US - UpScaled
